# Treatment with *Rhus tripartita* extract curtails isoproterenol-elicited cardiotoxicity and oxidative stress in rats

**DOI:** 10.1186/s12906-016-1318-3

**Published:** 2016-09-08

**Authors:** Abdelaaty A. Shahat, Mansour S. Alsaid, Syed Rafatullah, Mohammed O. Al-Sohaibani, Mohammad K. Parvez, Mohammed S. Al-Dosari, Vassiliki Exarchou, Luc Pieters

**Affiliations:** 1 Pharmacognosy Department (Medicinal Aromatic & Poisonous Plants Research Centre), College of Pharmacy, King Saud University, P.O. Box 2457, Riyadh, 11451 Saudi Arabia; 2 Phytochemistry Department, National Research Centre, 33 El Bohouth St. (former El Tahrir st.) 12622, Dokki, Giza, Egypt; 3 Pathology Department, KKUH, King Saud University, Po.box 2925, Riyadh, 11461 Saudi Arabia; 4 Natural Products & Food Research and Analysis (NatuRA), Department of Pharmaceutical Sciences, University of Antwerp, Universiteitsplein 1, 2610 Antwerp, Belgium

**Keywords:** *Rhus tripartita*, Anacardiaceae, Cardiovascular diseases, Isoproterenol, Oxidative stress, Chemical components

## Abstract

**Background:**

Consumption of plant-derived nutraceuticals and crude drugs in traditional medicine is widely believed to confer beneficial effects in thwarting the progression of cardiovascular diseases. *Rhus tripartita* (family Anacardiaceae) has been traditionally used to treat a wide range of ailments.

**Methods:**

In the present study we investigated the protective effects of an alcoholic extract of the stem part of *Rhus tripartita* male genotype (RTSM) on experimentally induced myocardial injury in rats. To this end, cardiac injury was induced by administration of isoproterenol (ISO) and serum enzyme markers, lipid profiles and cardiac tissue redox status were determined following RTSM treatment (250 and 500 mg/kg).

**Results:**

As a result, RTSM treatment significantly mitigated ISO-triggered upregulation of cardiac-specific markers of injury creatine kinase and lactate dehydrogenase. RTSM treatment significantly attenuated ISO-induced increase in serum cholesterol and triglycerides as well alterations in serum lipoproteins. Determination of oxidative balance showed that RTSM treatment significantly blunted ISO-induced increase in malondialdehyde and decrease in nonprotein sulfhydryl in cardiac tissue. Six compounds were isolated and identified as gallocatechin **1**, taxifolin **2**, myricetin-3-O-β-glucoside **3**, catechin **4**, epicatechin **5**, and 3′,8-binaringenin **6**. Compound **6** was isolated for the first time from the stem part of *Rhus tripartita*. Furthermore, RTSM treatment enhanced the survival fraction of cardiac cells exposed to oxidative stress in vitro.

**Conclusion:**

We conclude that the antioxidant properties of RTSM treatment underpin its cardioprotective pharmacological effects, thus, providing biological evidence for the treatment of cardiovascular diseases using *Rhus tripartita* in indigenous medicine.

## Background

Cardiovascular disease (CVD) is a major cause of morbidity and mortality in the modern era and includes conditions such as myocardial infarction, angina pectoris, and atherosclerosis [1]. Although several mechanisms are speculated to participate in the pathogenesis of myocardial insufficiency, the putative roles of these processes remain elusive. Mounting evidence suggests that deranged myocardial redox balance, enhanced inflammation and reduced cell survival influence normal heart function in CVD [2]. Herbal remedies have been utilized for various diseases including heart diseases [3, 4], as these herbs constitute a rich source of bioactive phytochemicals such as flavonoids, polyphenols and other constituents which have been shown to possess beneficial effects in CVD [5, 6]. The Anacardiaceae family of plants consists of trees, shrubs and/or woody vines belonging mainly to the genus *Rhus*, with about 250 species which occurs mostly in the tropics and subtropics and various temperate zones of the world [7, 8]. A number of *Rhus* species are used in folkloric and traditional medicine of many countries including Saudi Arabia. *Rhus tripartita* (Ucria) Grande is found wildly in the North-Eastern part of Saudi Arabia. In Arabian traditional medicine, different parts of *Rhus tripartita* plant have been used for centuries to treat inflammatory conditions as well as gastrointestinal and cardiovascular disorders [9]. The fruits of this plant are consumed freshly and in decoction to treat diarrhea and ulcers [10]. Previously, extracts and pure isolates of *Rhus tripartita* were isolated and shown to possess robust anti-inflammatory and antioxidant properties [11]. *Rhus* species were documented to possess a wide array of pharmacological activities such as anti-inflammatory, antinephritic, antimicrobial [12], antioxidant and breast cancer preventive properties [13, 14]. *Rhus coriara* (Sumac) was reported to be used for the prevention and treatment of atherosclerosis [15]. Recently, an attempt has been made to intensify the solvent extraction of total phenols and tannins from the bark of *Rhus tripartita*. From the same species of *Rhus*, flavonoids and isoflavonoids have been isolated from its aerial parts and characterized. Furthermore, butein (a polyphenolic component) was extracted from another species, *Rhus verniciflua*.

In our previous study on *crateagus* species, the phytochemical constituents (flavonoids and proanthocyanidins) and the therapeutic nature such as cardiovascular effects and antioxidant activity were conducted [16–18]. Our current research on Saudi Arabian *Rhus tripartita* showed that it is rich in flavonoids and proanthocyanidins compounds.

On account of its wide use in Arabian traditional medicine for the prevention of CVD, the present study, thus, explored the possible protective effect of an alcoholic extract of the stem part of *Rhus tripartita* male genotype (RTSM) against isoproterenol (ISO)-induced cardiac injury in rats.

## Methods

### Plant material

Fresh sample of stem bark of *Rhus tripartita* was collected in April 2013 at Hail area in the northwestern part of Saudi Arabia. The plants were identified by an expert Taxonomist at the Herbarium Unit. The voucher specimens have been deposited (SY 202/2013) at the Herbarium of the Faculty of Pharmacy, King Saud University, Riyadh, Saudi Arabia.

### Extraction and isolation

The stem of the plant was collected and air-dried at room temperature. The dried sample was powdered; 1100 g of dried sample was extracted with 4000 ml of 80 % aqueous methanol three times. The extracts were filtrated through Whatmann No. 1 and combined followed by concentration using a rotary evaporator under reduced pressure at 40 °C to yield a dry extract of 231 g (21 %). The percentage yield was expressed in terms of air dried weight of plant materials.

The dry extract (100 g) was diffused in 400 ml of distilled water and extracted successively with dichloromethane (CH_2_Cl_2_), ethyl acetate (EtOAc) and n-butanol (n-BuOH) (3 x 300 ml) respectively. Each extract was dried over anhydrous sodium sulfate. The organic fractions were concentrated under reduced pressure at temperature not exceeding 35 °C and the residual aqueous layer was lyophilized named (RTSM1), (RTSM2) (RTSM3) and (RTSM4) respectively. The EtOAc fraction (RTSM2) (20 g) was subjected to a Sephadex LH-20 column (Pharmacia) (90 x 4 cm), EtOH was used as a mobile phase. The fractions 50 ml each are collected and examined on TLC (Silica gel 60 F254, layer thickness 0.2 mm, Merck), the upper layer of mixture of EtOAc-HOAc-HCOOH-H_2_O (30/0.8/1.2/8) was used as a mobile phase. The TLC were viewed under UV (254 and 366 nm) before and after spraying with Neu’s spray reagent (reagent a) and vanillin H_2_SO_4_ (reagent b). Fractions 16-20, 21-35, 36-45, 46-60, 61-74 and 75-86 were combined together in one group and named as (RTSM2-I), (RTSM2-II), (RTSM2-III) and (RTSM2-IV) and (RTSM2-V) respectively.

The sub-fractions RTM2-II was subjected to Sephadex LH-20 column chromatography using methanol as eluent to give compounds **1** and **2**. Subfraction RTM2-III was subjected to Sephadex LH-20 using methanol as mobile phase to give compounds **3**, **4** and **5**. The sub fraction RTSM2-IV was subjected to column chromatography C18-reversed-phase with MeOH-H_2_O (6:4) as eluent to give compound **6**.

### NMR spectroscopy

NMR spectra were recorded in deuterated methanol (CD_3_OD) on a Bruker DRX-400 spectrometer (Bruker Biospin GmbH, Rheinstetten, Germany) operating at 400.13 MHz for ^1^H and at 100.61 MHz for ^13^C.

### In vitro cardioprotective assay

The H9C2 cells (human cardiomyoblast) were grown in DMEM medium, supplemented with 10 % heat-inactivated bovine serum (Gibco) and 1x penicillin-streptomycin (Gibco) at 37 °C with 5 % CO_2_ supply. The cells were seeded (0.5x10^5^ cells/well, in triplicate) in a 96-well flat-bottom plate (Becton-Dickinson Labware) and grown over night. Cardioprotective activity of RTSM was determined against 2, 7-dichlorofluorescein (DCFH; Sigma) cytotoxicity (IC_50_: 125 μg/ml), using MTT assay (MTT-Cell proliferation Assay Kit, Tervigen) as described previously [19] (Al-Yahya et al., 2016). Four doses of RTSM (31.25, 62.5, 125, and 250 μg/ml) were prepared in DMSO followed by dilutions in DMEM media. (>0.1 %, final). The cells were replenished with fresh media containing 120 μg/ml DCFH plus a dose of RTSM, including DCFH only-treated control. The cells were incubated for 48 h followed by MTT assay as per the manufacturer’s instruction. The optical density (OD) was recorded at 570 nm in a microplate reader (BioTek, ELx800). The cell survival fraction was determined by the non-linear regression analysis by Excel software, using the following equation:

$$ \mathrm{Survival}\ \mathrm{fraction}=\frac{OD\left[\mathrm{s}\right]-OD\left[b\right]}{OD\left[c\right]-OD\left[b\right]} $$

Where OD[s], OD[b] and OD[c] are the absorbance of sample, blank and negative control, respectively.

Also, a direct visual investigation was made under an inverted microscope (Optica, 40x and 100x) to observe any morphological changes in the cells cultured with different concentrations of RTSM and/or DCFH at 24 and 48 h.

### In vivo cardioprotective potential assay

#### Animals

The present study was approved by the Research Ethics Committee of the College of Pharmacy, King Saud University (Riyadh, Saudi Arabia). The handling of animals was in compliance with the Guidelines for the Care and Use of Laboratory Animals by the Animal Care Center. Wistar albino male rats, 180 ± 20 g were obtained from the Experimental Animal care Center of the college of Pharmacy, King Saud University, Riyadh. The animals were caged individually in hygienic conditions and kept in a controlled environment with a 12 h light-dark cycle at 22 ± 3 °C for a week before the experiment. The animals were offered free access to purina chow diet and water *ad libitum*.

### In vivo cardioprotective assay

Myocardial infarction (MI) induction in rats isoproterenol (ISO) (a synthetic catecholamine), is a well-accepted noninvasive drug to induce MI in rat model [20]. The physiopathological and morphological changes of ISO-induced myocardial necrosis are similar to those observed in humans [21]. ISO was dissolved in normal saline and injected subcutaneously (85 mg/kg b.w.) for two consecutive days at the interval of 24 h [20].

The animals were randomly divided into four groups, each containing six rats. Group I (normal group; control) animals received normal saline using intragastric tube for 14 days, and saline was administered (500 μl/rat, s.c.) on day 13 and 14 (24 h interval). Group II (ISO control; ISO) animals received normal saline for 14 days, and received ISO (85 mg/kg, s.c.) on day 20 and 21 (24 h interval). Group III (RTSM + ISO) animals received RTSM (250 mg/kg/day) orally for 21 days along with concurrent administration of ISO (85 mg/kg, s.c. at 24 h interval) on day 20 and 21. Group IV (RTSM + ISO) animals received RTSM (500 mg/kg/day) and ISO (85 mg/kg, s.c. at 24 h interval) on day 20 and 21.

### Estimation of marker enzymes

Levels of plasma alanine aminotransferase (ALT), aspartate aminotransferase (AST) [22], lactate dehydrogenase (LDH) [23], and creatine kinase (CK) [24] were then estimated usingReflotron® Plus Analyzer and Roche Diagnostic Kits (Roche Diagnostics GmbH, Mannheim, Germany).

### Estimation of lipid profile

The total cholesterol (TC) [25], triglycerides (TG) [26], and high-density lipoproteins (HDL-C) [27] were estimated in plasma using the Refloton instrument of the specific kits (Roche Diagnostics GmbH, Mannheim, Germany).

### Lipid Peroxidation (LPO) determination

The method reported by Utley et al. [28] was followed. The heart tissue was homogenized in0.15 M KCl (at 4 °C, Potter-Elvehjem type C homogenizer) to give a 10 % w/v homogenate. Aliquots of homogenate (1 mL) were incubated at 37 °C for 3 h in a metabolic shaker. Following this,1 mL of 10 % aqueous trichloroacetic acid (TCA) was added and mixed. The mixture was then centrifuged at 800 g for 10 min. Following this, supernatant (1 mL) was mixed with 1 mL of 0.67%thiobarbituric acid and placed in a boiling water bath for 10 min. The mixture was cooled and diluted with 1 mL distilled water. The absorbance of the solution was then read using spectrophotometer(UVmini-1240, Shimadzu Italia, Milano, Italy) at 532 nm. The content of malondialdehyde (MDA) (nmol/g wet tissue) was then calculated, by reference to a standard curve of MDA solution.

### Estimation of Non-Protein Sulfhydryl Groups (NP-SH)

Cardiac NP-SH was measured according to the method of Sedlak and Lindsay [29]. The heart was homogenized in ice-cold 0.02 M ethylene diaminetetraacetic acid (EDTA). Aliquots of 5 mL of the homogenates were mixed in 15 mL test tubes with 4 mL of distilled water and 1 mL of 50 % TCA. The tubes were shaken intermittently for 10 min and centrifuged at 3000 rpm. Two milliliters of supernatant were mixed with 4 mL Tris buffer (0.4 mol/L, pH 8.9) and 0.1 mL of 5,5′-dithio-*bis*(2-nitrobenzoic acid) (DTNB) and the sample was shaken. The absorbance was read within 5 min of addition of DTNB at 412 nm against a reagent blank.

### Determination of Total Protein (TP)

The TP was estimated by the kit method, supplied by Crescent Diagnostics, Jeddah, Saudi Arabia. The absorbance of this complex at 546 nm is proportional to the protein concentration. The serum total protein was calculated using the equation: Serum total protein = Abs. sample/Abs. standard × concentration of standard (1).

### Histopathological studies

The heart tissues were fixed in 10 % buffered formalin and processed using a VIP tissue processor. The processed tissues were then embedded in paraffin blocks and sections of about 5 μm thickness were cut by employing an American optical rotary microtome. These sections were stained with hematoxylin and eosin using routine procedures [30]. The slides were examined for *pathomorphological changes.

## Results

### 3′,8 Binaringenin (6)

NMR data:

^1^H NMR (CD_3_OD): δ 7.23 (1H, s, H-2’), 7.17 (3H, d, J = 8.1 Hz, H-2”’, H-6’, H-6”’), 6.88 (1H, d, J = 8.4 Hz, H-5’), 6.71 (3H, d, J = 8.1 Hz, H-3”’, H-5”’), 6.05 (1H), br s) and 5.89 (2H, br s, H-6,H-6”, H-8), 5.34 (1H, m) and 5.23 (1H, m) (H-2, H-2”), 3.02 (2H, m, H-3ax, H-3”_ax_), 2.79 (1H, m) and 2.56 (1H, m) (H-3_eq_, H-3”_eq_).

^13^C NMR (CD_3_OD): δ 197.0 and 195.5 (C-4, C-4”)166.9 and 164,7 (C-7, C-7”)164.0 (C-5), 163.5 (C-4”’), 163.2 (C-5”), 160.6 (C-4’), 157.2 (C-9), 155.2 (C-9”), 131.2 (C-6’), 129.8 (C-1’), 129.2 (C-1”’), 127.3 (C-2”’, C-6”’), 126.4 (C-2’), 120.0 (C-3’), 115.2 (C-3”’, C-5”’), 114.8 (C-5’), 105.6 (C-!”), 102.0 (C-10, C-10”), 95.6 and 95.0 (C-6, C-6”), 94.8 (C-8), 79.2 (C-2), 78.5 (C-2”); 42.6 and 42.2 (C-3 and C-3”).
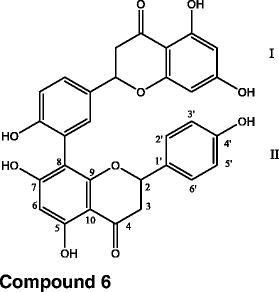


### In vitro cardioprotective potential of RTSM

The cardioprotective activity of RTSM against DCFH-induced injury on cultured C9H2 cells was investigated. While DCFH-toxicated cardiomyocytes were recovered to ~10 % with 62.5 μg/ml dose of RTSM, supplementation with 125 and 250 μg/ml of RTSM further enhanced the cardiomyocytes proliferation by ~15 and 30 %, respectively. Therefore, RTSM at the best minimal dose of 250 μg/ml showed the protective activity on cultured cardiomyocytes.

### In vivo cardioprotective potential of RTSM

To investigate the cardiovascular modulatory effects of RTSM treatment, we used the ISO-induced myocardial injury model in rats [31–33]. Firstly, we examined the effect of RTSM on ISO-induced changes in serum markers. To this end, administration of ISO (85 mg/kg) significantly increased serum levels of the enzymes SGOT, SGPT, GST and ALP, an effect significantly and dose-dependently thwarted by pretreatment with 250 and 500 mg/kg RTSM (Table 1). ISO administration further enhanced LDH and CK, cardio-specific serum markers of injury (Table 1). As shown in Table 1, pretreatment of rats with RTSM significantly attenuated ISO-elicited increase in serum LDH and CK suggesting the cardioprotective effects of RTSM treatment.

**Table 1:** Effect of RTSM on serum marker enzymes of control and experimental rats

Treatments	Dose mg/kg	SGOT(U/L)		SGPT(U/L)		GGT(U/L)		ALP(U/L)		LDH(U/L)		CK(U/l)	
		Mean ± SE	% change	Mean ± SE	% change	Mean ± SE	% change	Mean ± SE	% change	Mean ± SE	% change	Mean ± SE	% change
Control		79.00 ± 3.31		30.01 ± 2.44		4.85 ± 0.23		217.16 ± 11.14		106.08 ± 6.45		135.16 ± 5.43	
ISO only	85	200.00 ± 5.78***^a^		181.00 ± 7.33***^a^		13.65 ± 0.33***^a^		435.30 ± 10.02***^a^		165.66 ± 6.72***^a^		227.33 ± 10.18***^a^	
RTSM+ ISO	250	159.00 ± 6.53***^b^	21↓	150.83 ± 4.11**^b^	17↓	10.55 ± 0.40***^b^	23↓	379.33 ± 13.32*^b^	3↓	150.00 ± 4.91^b^	9↓	201.50 ± 5.51^b^	11↓
RTSM+ ISO	500	154.00 ± 4.01***^b^	23↓	140.00 ± 5.57**^b^	23↓	9.16 ± 0.25***^b^	33↓	350.33 ± 11.42***^b^	20↓	139.66 ± 6.19*^b^	9↓	185.50 ± 7.34*^b^	18↓

All values represent mean ± SEM. *p < 0.05, **p < 0.01, ***p < 0.001; ANOVA, followed by Dunnett’s multiple comparison test

^a^As compared with normal group

^b^As compared with only ISO only group

Experimental cardiac injury triggered by ISO is further associated with an alteration of serum lipid profiles [34]. To this end, determination of serum lipids revealed that administration of ISO significantly enhanced the serum levels of cholesterol, triglycerides, VLDL and LDL while significantly lowering serum HDL level (Table 2). As shown in Table 2, treatment of rats with RTSM significantly and dose-dependently counteracted ISO-induced alterations in serum lipids indicating a vascular protective role of RTSM treatment.

**Table 2:** Effect of RTSM on serum lipid metabolism and serum lipoproteins of control and experimental rats

Treatments	Dose mg/kg	Cholesterol(mg/dl)		Triglycerides(mg/dl)		HDL-C(mg/dl)		VLDL-C(mg/dl)		LDL-C(mg/dl)	
		Mean ± SE	% change	Mean ± SE	% change	Mean ± SE	%change	Mean ± SE	% change	Mean ± SE	% change
Control		98.1 ± 5.15		54.56 ± 3.45		42.66 ± 1.17		10.91 ± 0.69		44.52 ± 5.57	
ISO only	85	199.83 ± 7.21***^a^		155.00 ± 8.00***^a^		24.66 ± 1.56***^a^		31.001.60***^a^		144.16 ± 6.03***^a^	
RTSM+ ISO	250	179.83 ± 4.33*^b^	10↓	120.33 ± 3.44**^b^	22↓	27.66 ± 0.76	12↑	24.06 ± 0.68**^b^	23↓	128.10 ± 4.83^b^	11↓
RTSM+ ISO	500	157.83 ± 5.83**	21↓	105.61 ± 1.96***^b^	32↓	32.50 ± 1.17**^b^	32↑	21.12 ± 0.39***^b^	31↓	104.21 ± 6.57**^b^	28↓

All values represent mean ± SEM. *p < 0.05

**p < 0.01, ***p < 0.001; ANOVA, followed by Dunnett’s multiple comparison test

^a^As compared with control group

^b^As compared with only ISO only group

**Fig. 1:**
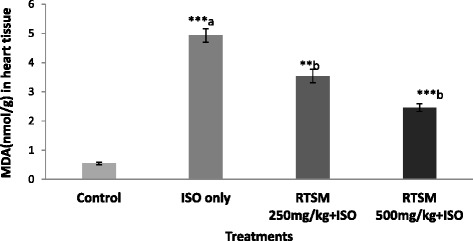
Effect of RTSM on the level of MDA in the heart tissue of the rats treated with Isoproteronol. All value represent mean ± SESM **P < 0.01, ***p < 0.001; ANOVA, followed by Dunnett’s multiple comparison test. ^a^As compared with control group. ^b^As compared with only ISO only groups

A further series of experiments addressed the possible role of redox sensitivity of RTSM treatment in ameliorating ISO-induced cardiotoxicity. To this end, we determined malondialdehyde (MDA) levels in cardiac tissue isolated from rats administered with ISO. As a result, ISO administration significantly upregulated tissue MDA levels, an effect that was significantly and dose-dependently mitigated by pretreatment with RTSM (Fig. 1).

**Fig. 2:**
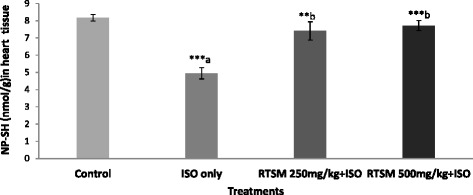
Effect of RTSM on the level of NP-SH (Nonprotein sulphydryl) in the heart tissue of the rats treated with Isoproteronol. All value represent mean ± SEM **P < 0.01, ***p < 0.001; ANOVA, followed by Dunnett’s multiple comparison test. ^a^As compared with control group. ^b^As compared with only ISO only groups

**Fig. 3:**
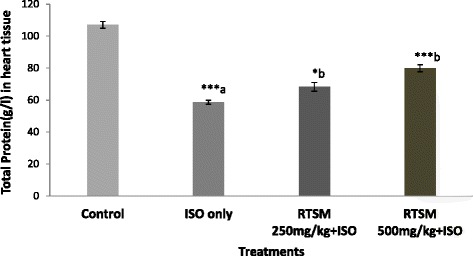
Effect of RTSM on the level of TP (Total Protein) in the heart tissue of the rats treated with Isoproteronol. All value represent mean ± SEM *P < 0.05, ***p < 0.001; ANOVA, followed by Dunnett’s multiple comparison test. ^a^As compared with control group. ^b^As compared with only ISO only groups

Additional experiments were performed to further elucidate the pivotal role of RTSM treatment in modulating the redox imbalance induced by ISO. ISO treatment is associated with decreased nonprotein sulfhydryl (NP-SH) levels in cardiac tissue [35, 36]. As illustrated in Fig. 2, administration of ISO significantly reduced NP-SH levels in cardiac tissue, an effect that was significantly and dose dependently blunted by pretreatment with RTSM indicating that the antioxidant effects of RTSM treatment are a critical mechanism in its cardio protective effect. Further examination of total protein levels in cardiac tissue revealed that ISO significantly decreased total protein levels, an effect that was significantly and dose-dependently counteracted by RTSM treatment (Fig. 3).

DCFH is generally used to measure in vitro oxidative stress generated by free-radicals through oxidation of DCFH to the fluorescent DCF [37].

**Fig. 4:**
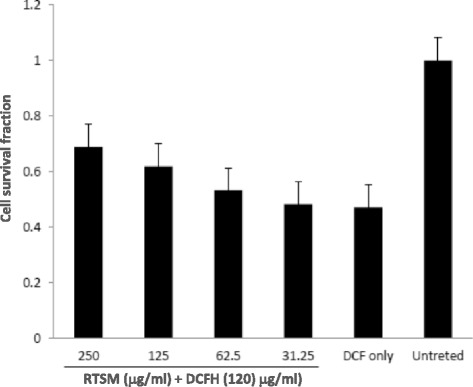
Cardioprotective effect of RTSM against DCFH-induced cytoxicity on cultured cardiomyoblast cells (H9C2) at 48 h post-treatment

To confirm the antioxidant effects of RTSM treatment in conferring a protective response against ISO-induced cardiac injury, we analyzed the survival fraction of cardiomyocytes ex vivo against DCFH -toxicity. As a result, survival fraction of cardiomyocytes treated with RTSM (125 μg/ml) was significantly enhanced by attenuating DCFH effect (Fig. 4).

**Fig. 5:**
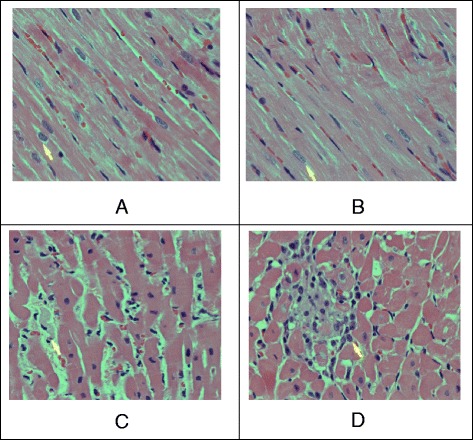
Photomicrography of cardiac tissue of rats showing the effect of RTSM. **a** Normal (control group) showing no significant pathology. H.& E. 400x; **b** Cardiac tissue showing myocardial necrosis with lymphohistiocytic, reaction around the necrotic myocardial fibers in rats myocardial cells treated with ISO only. H. & E. 600x; **c** RTSM (250 mg/kg) plus ISO pretreatment showing slightly disorganized myocardial fibres with focal chronic inflammation. H. & E. 600x. **d** RTSM (500 mg/kg) plus ISO pretreated group showing a small focus of myocardial fibres tic reaction. H. & E. 600x

### Histological improvement of injured myocardiac tissues by RTSM

The histology of rat cardiac tissues showed that ISO-elicited inflammatory lesions led to structure disorders of muscle fibers as well as infiltration of acute inflammatory cells, including extravasation of red blood cells (Fig. 5a and b). The other changes observed were interstitial edema and the appearance of vacuoles in ISO alone treated rats. RTSM treatment mitigated ISO-induced inflammatory changes in cardiac tissues, indicating that RTSM efficiently cured the tissue lesions in a dose-dependent manner (Fig. 5c and d).

## Discussion

Based on co-TLC, ^1^H and ^13^C-NMR and compared directly with published data, compounds **1**, **2**, **3**, **4** and **5** were isolated and identified before as gallocatechin **1**, taxifolin **2**, myricetin-3-*O*-β-glucoside **3**, catechin **4** and epicatechin **5**, respectively [6, 38, 39]. Compounds 6 was identified as 3′,8-binaringenin. The reported structure was confirmed by comparison with previously reported spectral data [39, 40]. Compound **6** was isolated for the first time from this species.

The present study reveals a, hitherto unknown, cardioprotective pharmacological effect of RTSM in an animal model of ISO-induced cardiotoxicity. Moreover, we show that oxidative stress that underpins ISO-induced cardiac injury serves as a prime target of RTSM in ameliorating pathological changes associated with ISO administration. Along those lines, RTSM significantly down regulated serum enzymes indicative of cardiac injury and significantly thwarted ISO-associated alterations in serum lipid profiles. Furthermore, RTSM treatment robustly mitigated ISO-induced alterations in cardiac tissue redox balance.

Mechanistically, the outcome of ISO treatment in rats comprises a myriad of biochemical alterations in the cardiac tissue [41]. ISO-elicited histopathological modifications are paralleled by oxidative and nitrosative stress resulting in the modification of redox enzymes such as superoxide dismutase, catalase and glutathione [42] as well as endogenous redox-sensitive mediators such as nitric oxide [43] and hydrogen sulfide. ISO-triggered generation of reactive oxygen species in cardiac tissue fosters enhanced apoptosis of cardiac cells [44] and is associated with increased cytosolic calcium, cAMP and mitochondrial depolarization [45]. Strikingly, these changes also involve endoplasmic reticulum stress in cardiac cells [46]. Ramifications of ISO treatment further include modulation of various signaling cascades including the activation of NF-kB [47] and mitogen-activated protein kinases [48]. It is tempting to speculate that these mechanisms participate in the amelioration of oxidative stress and cardioprotectivity conferred by RTSM treatment.

The antioxidant and cardiovascular beneficial effects of RTSM may be attributed to its bioactive phytochemical constituents. *Rhus tripartita* fruits were recently shown to contain a wide array of phytochemicals but were remarkably rich in flavones and betulinic acid [49]. Interestingly, flavones were previously reported to ameliorate ISO-induced cardiac injury [50] and hyperlipidemia [51]. Intriguingly, flavones were previously shown to alleviate ISO-induced increase in MDA levels [52]. Accordingly, our data show that RTSM attenuated ISO-induced increased MDA levels in cardiac tissue. DCFH is generally used to measure in vitro oxidative stress generated by free radicals through the principle of oxidation of DCFH to the fluorescent DCF [53]. In this study, we used DCFH because of its high toxicity on cultured cells. Our in vitro data showing cardioprotection against DCFH-induced injury was in line with the in vivo effects of RTSM in isoproteronol-myocardiac tissue damage in rats. Taken together, these results confirmed the cardioprotective potential of RTSM by using two different toxins in two different experimental models.

## Conclusion

In summary, sex compounds were isolated and identified. Compound 3′,8-binaringenin **(6)** was isolated for the first time from the stem part of *Rhus tripartita* RTSM treatment ameliorates experimental cardiac injury in rats effective, at least in part, by its antioxidant properties. Our study, thus, provides biological evidence for the cardiovascular beneficial effects of *Rhus tripartita* used in Arabian traditional medicine.

## Abbreviations

NP-SH: Nnonprotein Sulfhydryl
LPO: Lipid Peroxidation
TC: Total Cholesterol
TG: Ttriglycerides
HDL-C: High-Density Lipoproteins
MI: Myocardial infarction
CVD: Cardiovascular disease
DMEM: Dulbecco’s Modified Eagle’s medium
DCFH: 2,7-Dichlorodihydrofluorescein diacetate
OD: Optical Density
TCA: Trichloroacetic acid
EDTA: Ethylene diaminetetraacetic acid
DTNB: 5,5′-dithio-*bis*(2-nitrobenzoic acid)
VLDL: Very-low-density lipoprotein
LDL: Low Density Lipoprotein Cholesterol
